# Chitosan nanoparticles for the intracellular delivery of triphosphate nucleotide analogues

**DOI:** 10.1186/1471-2334-14-S2-P75

**Published:** 2014-05-23

**Authors:** Giovanna Giacalone, Elias Fattal, Hervé Hillaireau

**Affiliations:** 1 Galien Paris-Sud Institute, UMR 8612, Châtenay-Malabry, France

## Aim

Nucleotide analogues such as Azidothymidine-triphosphate (AZT-TP), the active form of Zidovudine, display an important pharmacological activity for the treatment of HIV. The administration of these nucleotide analogues would bypass the intracellular phosphorylation which can be a metabolic bottleneck. This possibility is however limited by their instability in physiological conditions, and furthermore their hydrophilicity restricts their access to the target cells. Several nanocarriers have been proposed so far for the encapsulation of these molecules, but their applications are limited due to the low drug loading achieved. Our strategy proposes the use of chitosan, a biocompatible and hydrophilic polysaccharide which is known to form nanoparticles through complexation with TPP; in contrast with previous methods, in our case the drug itself will be the driving force for the formation of nanoparticles [1].

## Material and methods

Different molar ratios between chitosan and AZT-TP (or ATP, used here as a model drug) have been tested in order to study the formation of nanoparticles; some selected ratios have been then evaluated for their size, surface properties, drug encapsulation and loading. In vitro cell uptake experiments were performed on a cell line of macrophages, using the free molecule as a negative control. The intracellular distribution of the delivered molecules was further investigated using confocal laser microscopy.

## Results

Colloidal suspensions have been obtained from chitosan and AZT-TP; nanoparticles present a minimal size about 200 nm with a zeta potential above +20 mV. An encapsulation efficiency of 70% can be reached, allowing loading rates as high as 44%. A cell viability of 80% has been found for particle concentrations up to 0.6 mg/mL. The cellular uptake is at least 2-fold higher when molecules are delivered as nanoparticles, compared to the free molecules.

## Conclusions

An original method is proposed to design nanoparticles, which allows high loading rates; this lowers the amounts of excipients needed, thus limiting the toxicity concerns. These nanosystems allow an efficient in vitro intracellular delivery of nucleotide analogues. Further in vivo studies will investigate the potential of these nanocarriers.
